# An automatic method to detect and track the glottal gap from high speed videoendoscopic images

**DOI:** 10.1186/s12938-015-0096-3

**Published:** 2015-10-29

**Authors:** Gustavo Andrade-Miranda, Juan I. Godino-Llorente, Laureano Moro-Velázquez, Jorge Andrés Gómez-García

**Affiliations:** Centro de Tecnología Biomédica, Universidad Politécnica de Madrid, Campus de Montegancedo, Crta. M40 km, 38, Madrid, Spain

**Keywords:** Glottis, Glottal Gap, Segmentation, HSDI

## Abstract

**Background:**

The image-based analysis of the vocal folds vibration plays an important role in the diagnosis of voice disorders. The analysis is based not only on the direct observation of the video sequences, but also in an objective characterization of the phonation process by means of features extracted from the recorded images. However, such analysis is based on a previous accurate identification of the glottal gap, which is the most challenging step for a further automatic assessment of the vocal folds vibration.

**Methods:**

In this work, a complete framework to automatically segment and track the glottal area (or glottal gap) is proposed. The algorithm identifies a region of interest that is adapted along time, and combine active contours and watershed transform for the final delineation of the glottis and also an automatic procedure for synthesize different videokymograms is proposed.

**Results:**

Thanks to the ROI implementation, our technique is robust to the camera shifting and also the objective test proved the effectiveness and performance of the approach in the most challenging scenarios that it is when exist an inappropriate closure of the vocal folds.

**Conclusions:**

The novelties of the proposed algorithm relies on the used of temporal information for identify an adaptive ROI and the use of watershed merging combined with active contours for the glottis delimitation. Additionally, an automatic procedure for synthesize multiline VKG by the identification of the glottal main axis is developed.

## Background

From the perspective of the laryngoscopic analysis, the voice production can be considered as normal when the left and right vocal folds present a symmetric oscillation. On the other hand, deviations from the symmetric pattern have been treated in the literature as clinical indicators of different vocal folds pathologies [1]. In particular, the cycle-to-cycle variability in the shape of the glottal waveform is found to be significant for most voice pathologies, and this variation is correlated with the perception of vocal roughness and breathiness [2]. However, there are certain levels of asymmetry that can not be considered pathological [3, 4]. Additionally, the literature reports different features extracted from the endoscopic video sequences that are capable of identifying in an objective way the presence of pathologies and evaluating the phonation process, i.e. the periodicity and amplitude of vocal folds vibration, mucosal wave, vertical level, glottal closure, phase closure, phase symmetry, presence of non-vibrating portions of the vocal folds [5, 6], etc. The evaluation of all of these features require first an accurate delimitation of the glottal gap. Thus, the glottal gap segmentation has been used as a previous step to improve the evaluation of voice disorders [7], also for assessing the quality of vocal folds vibration [8], and thus the effectiveness of the different treatments, medical or surgical.

There are two basic imaging procedures to capture the vibratory movement of the vocal folds: slow motion stroboscopy (SMS) [9] and high speed digital imaging (HSDI) [10]. The HSDI systems record images of the larynx at a typical rate of 4000 frames/s, while the rate obtained with SMS is only around 25 or 50 frames/s. HSDI illuminates using a continuous light whereas SMS uses a stroboscopic lamp to show the movement of the vocal folds taking advantage of the stroboscopic phenomenon. In the case of SMS videos, they also present an important intra-video variation and does not provide a real view of the vocal folds vibratory pattern, so its use is restricted to stable and periodic vocal fold vibrations [11]. In contrast, HSDI systems record every glottal cycle without temporal perturbation, being the only technique capable to register the true intra-cycle vibratory behavior of the vocal folds oscillations [12]. However, both methods present camera rotations, side movements of the laryngoscope, and artifacts due to movements of the patient, causing a delocalization of the vocal folds and the glottal gap (or glottis) that complicates the application of automatic image processing techniques [13].

Due to the large amount of data retrieved during the recording of these video sequences, the traditional assessment carried out in the clinical environment using both SMS and/or HSDI demands a large amount of human effort and expertise to manually process the recorded data. Despite, a human observer is very sensitive against static image properties such as shape, texture, color or scaling, he is unable to classify and remember the dynamic features in motion. For this reason, there are different methods that make use of glottal gap tracking in order to condense the time varying shape of the glottal area in a 2D representation. For instance: digital videokymograms (VKG) [14], glottal area waveform (GAW) [15] and phonovibrograms (PVG) [16].

**Fig. 1:**
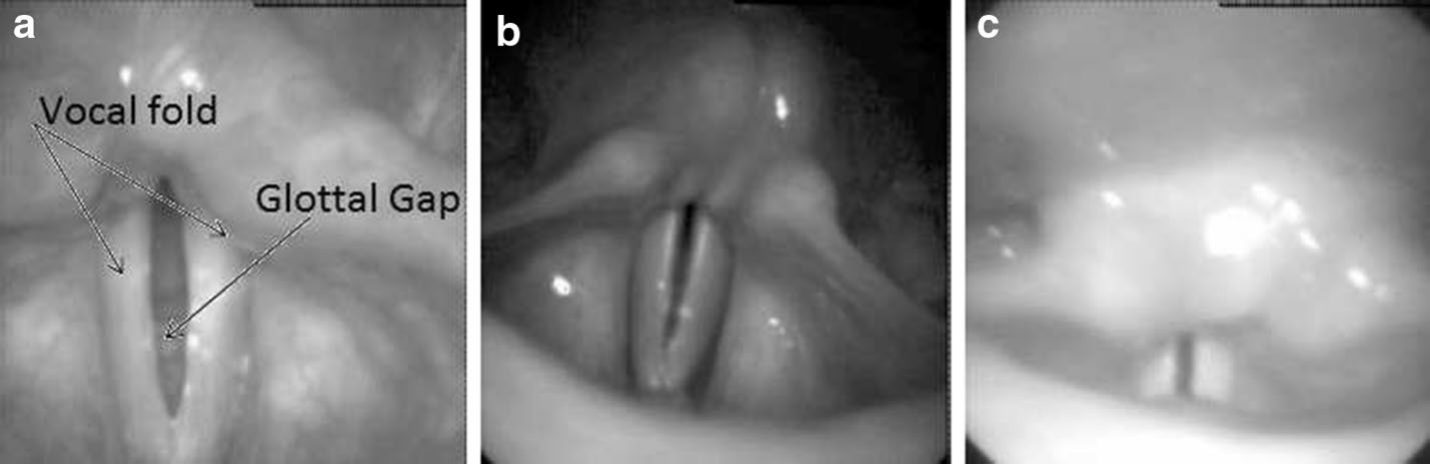
Different laryngeal images during phonation. **a** points out the vocal folds and the glottal gap; this video sequence presents a complete closure of the vocal folds. **b**, **c** present a glottal chink in the posterior part of the vocal folds which means that they do not have a complete closure. Additionally **c** has a occlusion in the anterior part of the glottis caused by the arytenoid cartilages. **a**, **b** and **c** show black artifacts due to reflections of the light inside the tube of the endoscope that carries the camera

In order to illustrate the difficulties of the glottal tracking, Fig. 1. shows three characteristic images of the larynx extracted from three different HSDI sequences. The position, orientation and size of the glottal gap significantly differ depending on the video. Moreover, the frames present different illumination conditions and black elongated areas originated by the recording equipment, specially in the corners and in the borders of the frames, as well as variations in the luminance of the glottal space. Additionally, some recordings present occlusion effects, meaning that the dominant parts of the vibrating glottis are hidden under the arytenoid cartilages or by other laryngeal structures (Fig. 1c).

The literature reports different techniques for the glottis segmentation task. Roughly speaking they can be grouped depending on the user intervention in semi-automatic and automatic methods. The semi-automatic techniques let the user interact as many times as needed in order to solve any inconvenience that might appear during the segmentation process. On the other hand, the automatic techniques process all the data without any previous setting or any user intervention. It is worth to emphasize that most of attempts found in the state of the art belongs to the second category [17–25]. From a clinical point of view, both methods present advantages and disadvantages that have been discussed before in the literature. Such discussion falls aside of the goals of this work, but it is worth mentioning that semi-automatic methods are more time consuming for the final user, although their accuracy is expected to be better.

With respect to the semi-automatic techniques, the literature reports different techniques and approaches. In [26] the user selects within the video sequence an arbitrary image and defines one or multiple seed-points belonging to the glottal area. A homogeneity criterion is defined via thresholding and interpolation for each line, then the user checks the first frame segmented and adapts the thresholds until a satisfactory segmentation is obtained. These thresholds are used to segment the whole video sequence. The authors report a segmentation time of approximately 5.3 s for 500 high-speed images. However, if the results are not according to the expectations the whole procedure must be repeated again. In [27] the posterior and anterior commissures are given by the user, and these regions are tracked along time using a block matching technique following the minimal gradient path from those points. They report a segmentation time for each frame of 0.04 s without considering the duration of the user intervention. In [28] the user defines the glottal midline by indicating the anterior and posterior commissure in the frame with the maximal opening; the user also defines a threshold based on a reference image to segment the HSDI. The segmentation time is not reported. In [29] the user searches around the video sequence for the frame with the minimal glottal opening which is used as an image reference for all the segmentation procedure. After, a binary difference is computed using the reference image and the isolated pixels are eliminated. Finally, a threshold value is chosen based on the lowest pixel value.

On the other hand, the automatic glottal segmentation of HSDI sequences has become an active research field [17, 27, 30] with growing interest in the last few years. The most common techniques reported in the literature are based on histogram equalization [28], region growing [19, 26], watershed [20] and active contours delineation methods [18, 21–23].

Histogram based algorithms are simple thresholding methods in which, given an image *I*(*x*, *y*), the task is to classify the pixels into groups by using a threshold. The glottis has darker intensity levels than the vocal fold tissues, so a threshold can be used to differentiate between the glottis and the vocal folds. However, the laryngeal images often have low contrast and heterogeneous profiles. Hence, selecting a global threshold results in an erroneous delimitation of the glottal gap, since the intensity distribution is not bimodal.

Region growing methods start selecting a number of seed pixels. Each of these seeds is regarded as a region. Later, the algorithm adds to the regions the neighboring pixels with similar image features, thereby growing the regions. The choice of the homogeneity criterion is crucial for the success of the algorithm. The issues with the region growing are that it requires a solid criterion for the seed selection and relatively well delimited edges in order to converge towards the glottal space. Furthermore, the algorithm segments objects with inhomogeneous regions into multiple sub-regions, resulting in over-segmentation. In [19] the authors combine the use of histogram thresholding with region growing: first they initialize the seeds using advanced thresholding techniques based on the histogram of the images; then the seeds are used for the region growing algorithm.

The watershed simulates a rain over the image where each pixel represents an altitude as a function of its grey level. The drops that fall over a point flow along the path of steepest descent until reaching a minimum. Such a point is labeled as belonging to the reception basin associated with this minimum. The result of the watershed are thousands of catchment basis leading to an over-segmentation. This algorithm is used in [20] for SMS images which have a much better contrast than HSDI. However, in most of the laryngeal images the watershed does not eliminate all the artifacts that do not belong to the glottis, making necessary an additional classification step in order to remove those objects causing loss of glottal information (i.e when the glottis is divided in two or even in three parts).

Lastly, the active contour algorithms or snakes are popular in medical images since they can be coupled appropriately to non-rigid and amorphous contours by an iterative minimization of a energy function. The authors in [31] propose an active contour algorithm for the extraction of the vocal folds from HSDI, adapting a linear filter to a Canny edge detector to reduce the noise and to calculate the external energy. The initialization is performed using the results obtained from previous frames, selecting empirically the parameters for the internal and external energies. In [18] a local region-based framework for guiding the active contours is used. The foreground and background are modeled in terms of small regions as constant intensities represented by their means, allowing to split or merge the active contour. However, the initialization process always assumes that the glottis has a vertical orientation. Some of the aforementioned algorithms do not consider that the glottis corresponds to less than 25 % [18] of the area of the image, and they do not take into account the temporal dimension of the problem, so each frame is treated individually, leaving aside the information obtained from the previous frames.

Having the above mentioned facts in mind, a complete automatic framework is proposed in this paper to track the glottal gap along time using data from HSDI recordings. The method is based on analyzing the row and column intensity variations throughout the video sequence to identify the region with the highest rate of change, which is considered as the region of interest (ROI). The identification of the ROI reduces the number of false detections, the computational burden, and can be iteratively updated to be tolerant for camera displacements. Moreover, the identification of the ROI opens the possibility to locate the glottis using a more simple method, such as the one proposed in [20], just by correlating with a standard template obtained empirically. In this way, the method proposed provides a robust initialization for each frame, making the process fully automatic. Finally, in order to refine the segmentation, the proposed procedure uses an algorithm based on active contours [32]. The paper is organized as follows. “Methods” details the methodology proposed. “Results” explain the setting procedure for the different parameters and the results obtained based on a database of high-speed videos. Finally, “Discussion” presents some conclusions and discussions.

## Methods

**Fig. 2:**
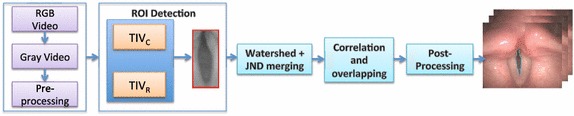
Proposed framework. Graphic representation of the different steps followed to segment the glottal gap: pre-processing, ROI detection, watershed first region merging, correlation merging, and post-proccessing

This section describes the framework proposed for the automatic tracking of the glottal gap. Figure 2 summarizes graphically the different steps of the process. In the following subsections the different steps of the procedure are detailed.

### Pre-processing

In spite of the glottis is the darkest object found in the images extracted from the videoendoscopic sequences, the surrounding tissue of the vocal folds and the glottal gap are usually difficult to differentiate due to the illumination conditions and low image contrast. Modeling the histogram of a HSDI with a statistic distribution, such as Rayleigh as in [19], or finding the darkest region, produces in errors due to the non-uniform contrast of the image, lighting conditions and artifacts due to the recording equipment. For this reason, it is required to simultaneously reduce the effect of the low contrast and to highlight the object of interest (i.e. the glottis). Thus, the use of image enhancing techniques is expected to improve the characteristics of the image for a further processing.

The literature reports the use of different enhancing techniques as a previous step to the application of the segmentation algorithms. In [24], the authors combine an anisotropic diffusion with a FFT based band pass filter, in order to obtain a smoother image without losing edge information.

In [23], the authors use a global thresholding to obtain a binary image to eliminate the worthless information, However this strategy can not be generalized for noisy and poor quality HSDI laryngeal recordings. Another alternative is to manipulate the histogram of the image. The most common histogram based processing techniques are the histogram equalization, adaptive histogram equalization (AHE), contrast limited histogram equalization (CLHE), and the contrast limited adaptive histogram equalization (CLAHE). CLAHE is used in [18] providing more details in the glottal area while avoiding significant noise introduction. CLAHE highlights the details over a small neighborhood preventing the over amplification of noise that can arise from adaptive histogram equalization (AHE).

One of the most widespread methods is based on point-wise intensity transformations. The point-wise transformation operates directly on the intensity values of an image, processing each pixel separately and independently. This transformation can be linear, piecewise linear, or non-linear. Aghlmandi and Faez [25] establish a methodology for pre-processing SMS images as a previous step for edge detection. The authors mention the drawbacks that exist in the acquisition due to the flashing effect at the recording instants, reducing the accuracy of the segmentation algorithm. In order to solve these issues, they proposed the use of a non-linear transformation ($I_{out}$) as follows:

1 $$\begin{aligned} I_{out}(x,y)= {\left\{ \begin{array}{ll} 255 &{}\quad \text {for } I(x,y) >L_i\\ 255 \times \left( \frac{I(x,y)}{L_i}\right) ^\zeta &{}\quad \text {for } I(x,y)\le L_i \\ \end{array}\right. } \end{aligned}$$

$$\begin{aligned} L_i=\frac{1}{m\beta }\sum \limits _{j=1}^m I(x_j,y) \end{aligned}$$

where *I*(*x*, *y*) denotes the stroboscopic image; $L_i$ is the mean of lighting levels in each row *i* of the image; *m* is the number of columns in the image; $\beta$ is an adjustable factor for increasing or reducing the contrast (set to 1.3); and $\zeta$ is a coefficient that is set to 1.8. The same pre-processing is used in [22] to highlight the glottal area and to reduce the influence of the flashes in HSDI. The $\beta$ parameter is crucial to improve the contrast, since wrong values produce results in which is hard to distinguish between the glottis and the surrounding tissues or in other cases loss of glottis information. The decision of which $\beta$ is the best option to enhance laryngeal images is depend on the trade-off between contrast and information loss. In this paper the non-linear transformation is chosen for the pre-processing step, the justification and the selection of the parameters are evaluated in the “Results” section.

### ROI localization

The ROI detection permits to eliminate more than 25 % of the non-relevant information found in the laryngeal images, so it is an important step to be considered prior to the segmentation process. The literature reports some attempts to detect a ROI, however most of these works require previous knowledge about the configuration of the HSDI, need the user intervention and, even more important, they do not consider the temporal information of the sequence. In [22], the authors assume that the segmented glottal area from the previous frame is available, and set to 1 the values of the pixels for which the difference between the analyzed frame and the previous is larger than 20 % of the maximum value of the image. Another algorithm based on differences between consecutive images is proposed also in [29]. In [26] an user selects an initial seed in an frame with the vocal folds open. This can be understand as a ROI, since the region growing starts its journey from such a manual initialization. The method reported in [18] is an edge-based morphological processing of some frames extracted from the HSDI, called landmarks. The landmarks are a set of frames that represent the open phase of the glottal cycles within the sequence under analysis. The idea of the morphological operator is to find a large, nearly vertically oriented area and to apply a Sobel filter to detect the strong edges in the vertical direction. Then, a morphological closing operation is carried out over the gradient map to connect small related regions. The regions to be connected are identified by means of connected component analysis. Lastly, the object with the largest area and vertical orientation is chosen. Around the selected area, a rectangle is delineated, representing the ROI.

**Fig. 3:**
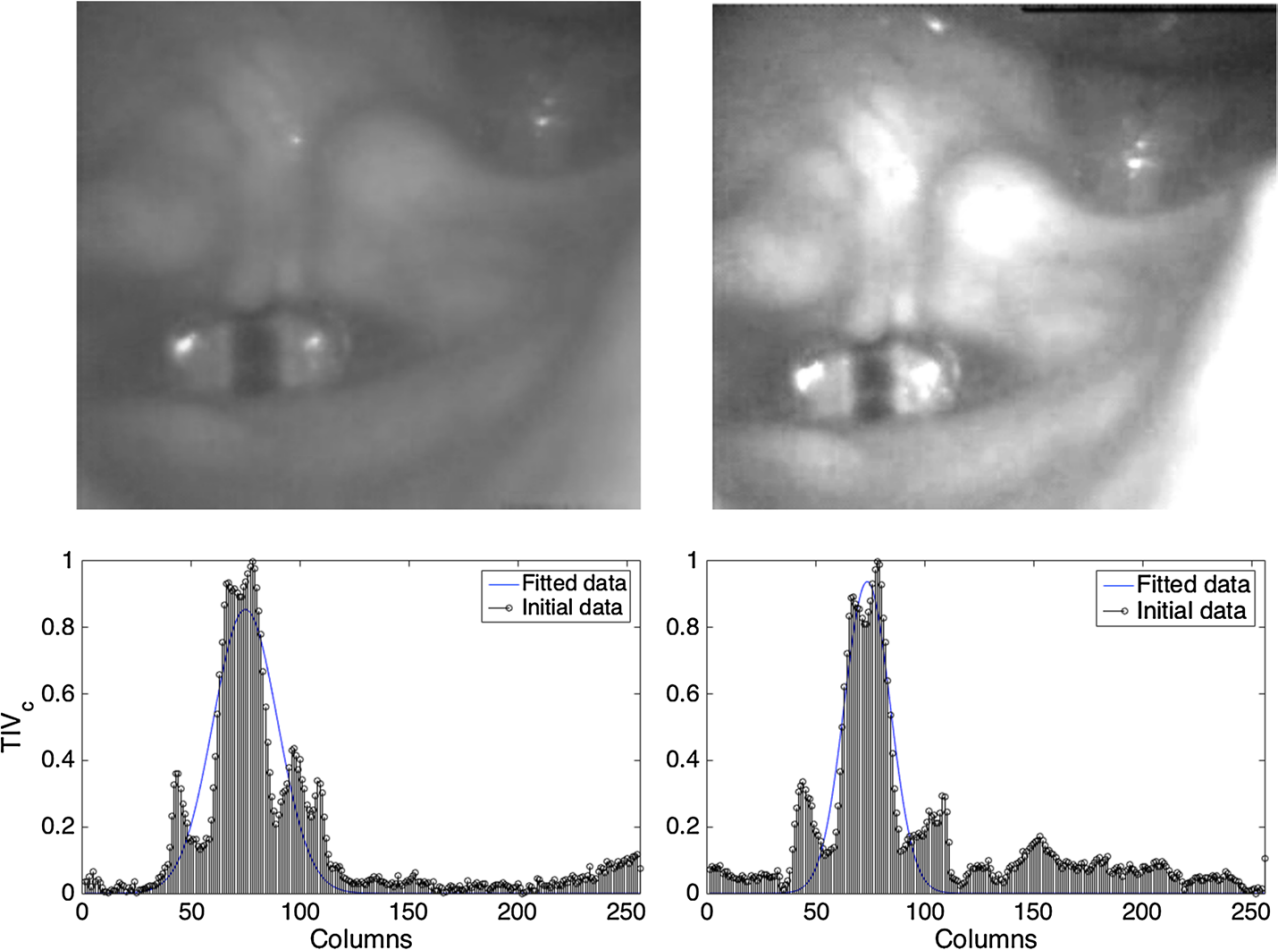
$TIV_{c}$ for 100 frames. The first image corresponds to a $TIV_c$ without pre-processing and the second to a $TIV_c$ with pre-processing. The *black bins* represent the original $TIV_c$ and the *blue curve* is the fitted Gaussian version

Since the displacements of the glottis are small between consecutive frames, images taken at consecutive time instants are strongly correlated among them. Thus translation movements in a short period of time are almost null. However, due to the involuntary movements of the camera or the patient, or due to the skills of the clinicians during the acquisition, the recordings present small displacements of the focus that are more significant as the number of evaluated frames increase. Considering the aforementioned, establishing a criterion based on the change of the spatial intensity profile to detect the ROI each *N* frames is a good choice. The squared area to be tracked is selected adaptively based on the variations of the image intensity and the inter-frame disparity for an appropriated set of frames, reducing the effect of the transversal shifts. By taking advantage of the continuous light source used to record the HSDI, the area with the largest variability within the image can be identified. This is done by analyzing the cumulative intensity variation of each frame in the *x* and *y* coordinates. When delineating the ROI, it is also important to consider the periodic reflections (highlights) that could appear in the image and that would increase the size of the ROI. However, the non-linear transformation done in the pre-processing step mitigates the influence of flashes as it has already been demonstrated in previous studies [22, 25] and it shows also in Fig. 3. In the end, the area with the highest variability along time is identified as the glottis.

In order to do so, firstly, it is necessary to convert the original RGB video sequence to a grey scale through a transformation according to the model YIQ [33]. After such conversion, the luminance Y, is used to generate the new video sequence in the grey scale. Finally, the total intensity variation in columns ($TIV_c$), and the total intensity variation in rows ($TIV_r$) are computed. The steps that describe this novel approach developed in this work are explained in the following subsections.

#### Total intensity variation in columns ($TIV_c$)

The first intensity variations to be analyzed are those related to the columns of the laryngeal images. The reason for starting the analysis in the columns stands on the fact that the main axis of the glottal gap is usually located in a quasi-vertical position (with a slope of more than 30 degrees with respect to the horizontal axis). Hence, the information arising from the cumulative intensity variation in the horizontal axis is more significant than in the vertical one. In order to obtain the total intensity variation in columns $(TIV_c)$, it is necessary to define two additional terms: the intensity variation matrix $S_{c}(x,t)$ and the average intensity variation vector ($AIV_c$). In $S_{c}(x,t)$, each row represents the intensity variation of the columns for each frame. The (2) describes the mathematical procedure to compute $S_{c}(x,t)$.

2 $$\begin{aligned} S_{c}(x,t)=\frac{\sum \nolimits _{y=1}^nI(x,y,t)}{n} \begin{array}{c} \qquad \forall x, 1\le x \le m; \\ \qquad \forall t, 1\le t \le N; \end{array} \end{aligned}$$

where *I*(*x*, *y*, *t*) is the HSDI video sequence with its respective *x*, *y* and *t* coordinates, *n* and *m* are the number of rows and columns of each frame respectively. Lastly, *N* is the number of frames that are used to find the ROI, this value is adjustable with a value not exceeding the maximum number of frames in the video.

The average intensity variation $AIV_{c}(x)$ (3) is a vector, in which each of its elements represents the horizontal intensity variation for the *N* frames evaluated. Finally, the total intensity variation in columns $TIV_c$ (4) is computed through analysis of the intensity variation of each frame with respect to the average intensity variation of the *N* frames by means of the Mean Absolute Error (MAE). For the ROI problem the most interesting points are those reporting the highest error.

3 $$\begin{aligned} AIV_{c}(x)=\frac{\sum \nolimits _{t=1}^NS_{c}(x,t)}{N}\qquad \forall x, 1\le x \le m; \end{aligned}$$

4 $$\begin{aligned} TIV_{c}(x)=\frac{\sum \nolimits _{t=1}^N|S_{c}(x,t)-AIV_{c}(x)|}{N}\quad\forall x, 1\le x \le m; \end{aligned}$$

The (4) represents the region with the largest variability in the *N* frames under consideration, and its behavior resembles to a Gaussian-like function whose center coincides with the main axis of the glottal gap (Fig. 3). In order to obtain the cut-off points on the *x*-axis, $TIV_c$ is fitted to a Gaussian distribution $\mathcal {N} (\mu ,\sigma ^2)$ using the Non linear Least Squares method [34]. The mean of the gaussian will be the column with the greatest intensity change and the standard deviation will determine the size of the ROI. The cut-off points in the *x*-axis are obtained using (5).

5 $$\begin{aligned} X_{cl}=\mu _x-\kappa _x\sigma _x; \qquad X_{cr}=\mu _x+\kappa _x\sigma _x; \qquad TI_{X}=[X_{cl},X_{cr}] \end{aligned}$$

where $X_{cl}$ and $X_{cr}$ are the left and right cut-off borders respectively, $\kappa _x\sigma _x$ is the standard deviation and $TI_{X}$ is the tolerance interval that indicates the width of the ROI in the *x*-axis. The procedure described above results in a new sequence $I_c(x,y,t)$ which is a bounded version of the original one.

#### Total intensity variation in rows ($TIV_r$)

**Fig. 4:**
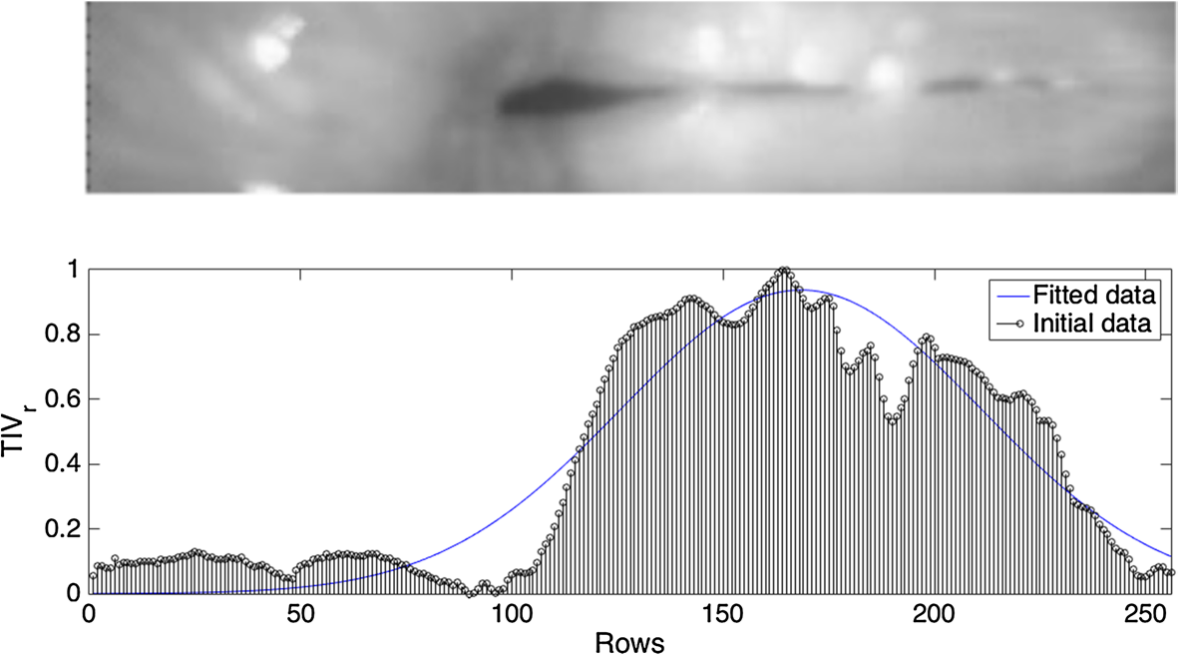
$TIV_{r}$ for 100 frames. The video sequence presents a glottal chink and the glottis is split into two, illustrating one of the most demanding cases. The HSDI is rotated in horizontal position for a better visualization

The total intensity variation in rows $TIV_r$ is computed following the same criteria used previously for $TIV_c$ but with slight differences, since $TIV_r$ uses the reduced area obtained in the previous step as a starting point, and further evaluates the variation in rows. The $TIV_r$ computation deals with two complex scenarios; the first is when the glottis is divided in two or more regions. This problem does not affect the normal performance of the ROI detection despite of the presences of extra valleys in the $TIV_r$ since an average movement is computed for *N* frames reducing the effect of the valleys. The second scenario is even more demanding and corresponds to the presence of glottal chink. Here, depending of the up and down cut-off points in the *y* axis ($Y_{cu}$ and $Y_{cd}$), some information in the posterior part could be lost. Nonetheless, this scenario does not commit the general performance of the algorithm since there is a optimal range for no loss of information as it will be show in “Results” section. Figure 4 shows a HSDI in which both scenarios are presented.

#### Adaptive ROI for motion compensation (MC)

The displacements of the endoscope affects the alignment of the HSDI image pixels along time, leading to difficulties to track the dynamic characteristics of the laryngeal structures. Thus, methods to compensate these displacements are needed. In [13], Dimitar argues about the importance of a procedure to compensate for the distortion originated by the endoscope displacements in the synthesis of a VKG, which causes intermittent changes of the voicing pattern and difficult the interpretation and further analysis. Note that the movement of the vocal folds (70–400 Hz) is much faster than the one originated by the endoscope ($\sim$15 Hz), so the motion caused by the endoscope is indistinguishable in one glottal cycle. In order to clarify this, consider the case of the vocal folds vibrating with a fundamental frequency $f_o=$ 100 Hz (period $T_o$, equal to 10 ms) and an endoscope displacement with a frequency of $f_e=$ 15 Hz (period $T_e$, equal to 66.67 ms). In this scenario the movement of the endoscope would be significant considering at least 6 glottal cycles. With this in mind, the implemented ROI detection methodology recomputes the region of interest every *N* frames so, this value is set up to compensate the camera motion, thus reducing the false detections.

The value of *N* is undoubtedly one of the most important parameters of the proposed methodology to accurately detect the ROI and for the motion compensation. This parameter could take any value between 1 up to the total number of frames. However a value close to 1 limits the possibility to characterize the motion that is present. Contrariwise, if the value of *N* is close to the total number of frames, non valuable information is added to the ROI increasing the false detections. Additionally, *N* provides reliability against the camera and/or patient displacement. With a small value of *N* the algorithm becomes more robust against movements, avoiding the effects of those displacements that are related with the endoscope.

### Watershed transform and first region merging

After reducing the size of the area to be analyzed, the next step is the identification of the glottis boundaries. In this work, the algorithm used for this purpose is the one described in [20], which is based on a watershed transform combined with a Just Noticeable Difference (JND) based region merging. The watershed transform creates a set of well delimited objects, opening the possibility to identify their individual features and statistics to find out those that belong to the searched object. Nevertheless, the results of the watershed transform are usually disappointing, due to the fact that thousands of objects arise when only a few were expected. This problem is called over-segmentation and is mainly due to noise in the image. There are two ways to reduce the over-segmentation; pre-processing the gradient image with thresholding, or a post-processing by merging objects with one or more features in common [35, 36]. A thresholding with a value of 10 is applied to the normalization of the image gradient and those pixels with a grey level below 10 are assigned to 0 so, they are converted into a minima that can only belong to the internal part of any region. This simple thresholding removes most of the regions that appear due to the intrinsic noise originated by the HSDI acquisition and the ones produced for the tissues texture. The threshold applied to the gradient image has been chosen to avoid removing significant edges of the image. On the other hand, the merging criteria is based on a fixed threshold over a cost function that decides if two regions are to be merging or not. The differences between the methods used in this point lie on the definition of the merging cost function. The chosen cost function is calculated using the JND of different grey levels of the image and has been theoretically defined in [37]. JND is a quantitative measure for distinguishing the luminance changes perceived by the human visual system. In other words, JND gives the maximum difference of the luminance values that can be differentiated by the human eye. The function for evaluating the visibility threshold of the JND is described by the equation:

6 $$\begin{aligned} JND(k) = {\left\{ \begin{array}{ll} D_0=[1-(k/127)^{0.5}]+3 &\quad k \le {127}\\ \gamma (k-127)+3 &\quad \text {otherwise} \end{array}\right. } \end{aligned}$$

where *k* is the luminance value within [0, 255], and the parameters $D_0$ and $\gamma$ depend on the viewing distance between a tester and the monitor. $D_0$ denotes the visibility threshold when the background is 0 and $\gamma$ denotes the slope of the line that models the JND visibility threshold function at higher background luminance. The values of $D_0$ and $\gamma$ are set to 17 and 3 / 128, based on the subjective experiments done in [38]. The merging cost function used to fuse the region is computed by the equation:

7 $$\begin{aligned} \begin{aligned} F_c=[|mR_1 -mR_2| - \min JND(mR1,mR2)+255]\\ \end{aligned} \end{aligned}$$

where *mR*1 and *mR*2 are the average values of the grey level of each neighbor region, and *minJND* is the minimum JND between the average of both regions. The goal of the merging cost function is to combine all the regions in which $F_c$ is below an specific threshold *Thr*. Those regions with an average grey level below *Thr* are said to belong to the same region. Based on the experimentation and considering the thresholding used in [20] for SMS videos the *Thr* value is set to 265. Additionally, the JND function is slightly modified in order to reduce the brighter regions. This is done by firstly finding the frame with the maximal glottal opening inside the *N* ROI frames, $I_{{ROI}_{t=min}}$, which corresponds to the photogram with the minimum intensity value and with the highest glottal gap opening (8). The intensity distribution of the glottis and background in $I_{{ROI}_{t=min}}$ has a cuasi-bimodal behavior. Considering this fact, it is feasible to reduce part of the meaningless information by Otsu’s method [39]. Since this automatically performs a clustering-image thresholding assuming that the image contains two classes of pixels (glottis and background). For all the values over the Otsu’s threshold a JND of *Thr* has been assigned so the bright regions of the image (background) belong to an unique region and the amount of information is drastically reduced. However, there is still over-segmentation caused by the intensity disparity inside the glottis and also by the presence of regions that despite not being part of the glottis, they have some of their intensity features.

8 $$\begin{aligned} I_{{ROI}_{t=min}}=\underset{t=1..N}{{\text {argmin}}}\Bigg ({\sum _{x}\sum _{y}I_{{ROI}_{t}}(x,y)}\Bigg ) \end{aligned}$$

### Correlation regions merging

**Fig. 5:**
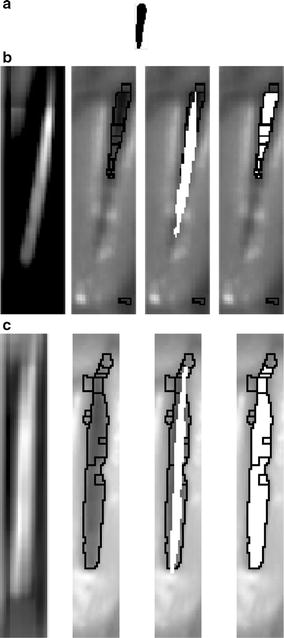
Merging steps. **a** Standard template found empirically based on manual segmentation; **b**, **c** show from *left* to *right*: two different frames of two different sequences, similitude matrix, first region merging, cross-correlation overlapping and correlation region merging

The next step consists of another merging process. The goal now is to join all the regions that correlate with a standard template obtained from the database. The standard template was obtained empirically based on manual segmentations carried out by one expert in all the frames of the database with the maximal glottal opening. The potential templates are built with white background and a black foreground. The white background acts like an edge enhancer in order to highlight the glottis contour. The test involves the use of different glottis shapes, resizing, warping and small rotations. After an intensively evaluation of all of these features, a standard template that better correlates with the available data is obtained. The standard template resizes automatically depending of the ROI size, ensuring not be affected by different zoom of the glottis. The standard template obtained for a ROI of 40 × 148 has a size of 12 × 42 (see Fig. 5a). This template is used as a baseline for the correlation merging.

The standard template is correlated with each frame using the normalize correlation coefficient, providing values within the range $[-1, 1]$. If both images are absolutely identical the value is 1; if they are completely uncorrelated, 0; and if they are completely anti-correlated, $-$1 (for example if one image is the negative of the other). The normalize correlation coefficient has been selected due to its invariance with respect to the intensity and because its similitude matrix provides valuable information about the glottis and vocal folds position. The threshold for a good matching is established in 0.45 in the similitude matrix. Below this value the glottis is considered fully closed. The regions obtained by the cross-correlation are intersected with the results of the first merging process, and the overlapped objects are merged. Figure 5b, c show the complete correlation and merging procedure carried out to detect the glottis. The first image represents the similitude matrix; the second represents the first region merging; the third one the overlapping between the cross-correlation and the first region merging; and, finally, the fourth represents the results of the second merging process. However, due to the inter-video variability the merging process could not be enough to obtain a reliable glottis segmentation. In order to clarify this idea, the last image in Fig. 5b represents an example in which the glottis presents an irregular shape and it is not well delimited in the anterior part. This phenomenon appears due to the difficulties of establishing an unique criterion for the region merging (threshold of the cost function) even when there exists large inter video changes in the illumination conditions. For that reason a post-processing stage is required to refine and smooth the segmentation obtained from the second merging region.

### Post-processing: localizing region-based active contours

**Fig. 6:**
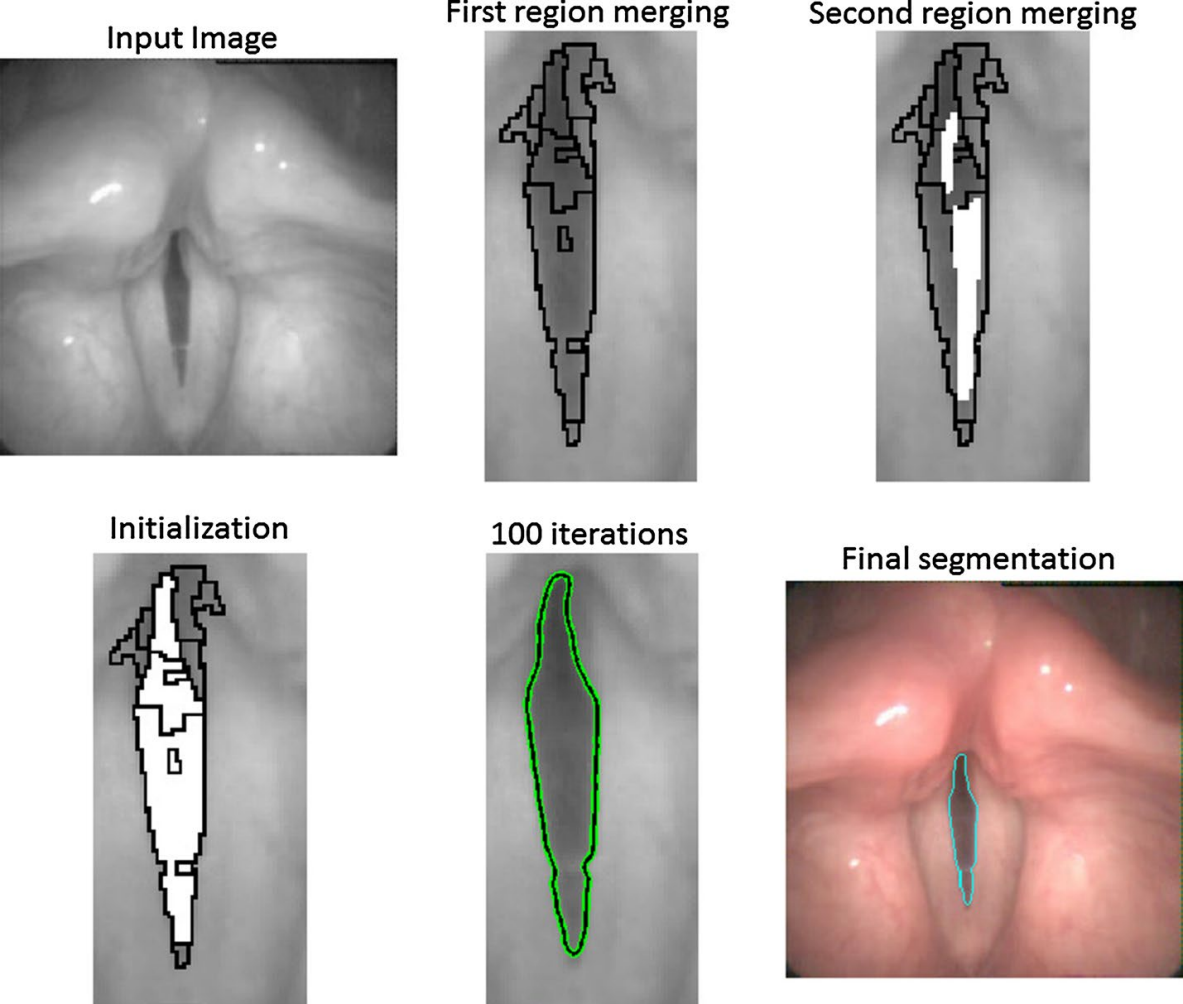
Complete methodology representation. From *top* to *down* and *left* to *right*: Input image; segmentation obtained after watershed and first region merging; second region merging (the *white part* of the image represents the region that correlates with the previous step); overlapping results and initialization for the active contour; and final delimitation of the glottis after 100 iterations

There are two main categories for the active contour models or snakes: edge-based and region-based. The edge-based image gradients are used to identify the object’s boundaries. The main limitation of this model is that it usually incorporates the edge information ignoring other image characteristics. The second disadvantage is that it must be initialized close to the local minima of interest in order to avoid the snake to be trapped in other local minima. Meanwhile for the region-based models, the foreground and background are described statistically and this model tries to find the energy that best fits the image. The advantages of this technique include robustness against initial curve placement and insensitivity to image noise. However, techniques that use global statistics are usually not ideal for segmenting heterogeneous objects. In cases where the object to be segmented cannot be easily distinguished in terms of global statistics, region-based active contours may lead to erroneous segmentations. Glottis detection in laryngeal images has a certain degree of complexity because these images are heterogeneous and noisy at the same time. Heterogeneity and noise can be solved using the local statistics approach proposed in [32]. The idea is to model the foreground and background in terms of smaller local regions, since foreground and background regions cannot be always represented with global statistics. This framework allows a correct conversion in cases of inhomogeneity, common in medical images. The analysis of local regions leads to the construction of a family of local energies at each point along the initial curve. In order to optimize the local energies, each point of the curve is considered separately and moves to minimize the energy computed in its own local region. The energy can be modeled in three different ways: the uniform modeling energy, the means separation energy, and the histogram separation energy. In this paper, the Chan-Vessel model was chosen, which models the interior and exterior of the region as constant intensities represented by their means. The experimentation carried out has shown that the anterior and posterior part of the glottis are not always accurately segmented during the correlation merging step, producing in some cases a wrong delineation of those regions. The post-processing uses the result of the correlation regions merging as initialization for the snake. For instance the first figure of the second row in Fig. 6 represents an example of initialization used for the active contour, showing that the anterior part is not segmented correctly by the watershed and the correlation merging.

Since the post-processing step is only a refined version of the previous steps, the number of iterations of the active contour and the radius of the local region can be fixed without an extensive analysis of the database. The radius determines how local the resulting segmentation are, therefore a radius of 5 pixels is enough for a refined procedure. The number of iterations is set to 100 ensuring full convergence in the glottis.

### Glottal main axis localization with application to the automatic synthesis of VKG

**Fig. 7:**
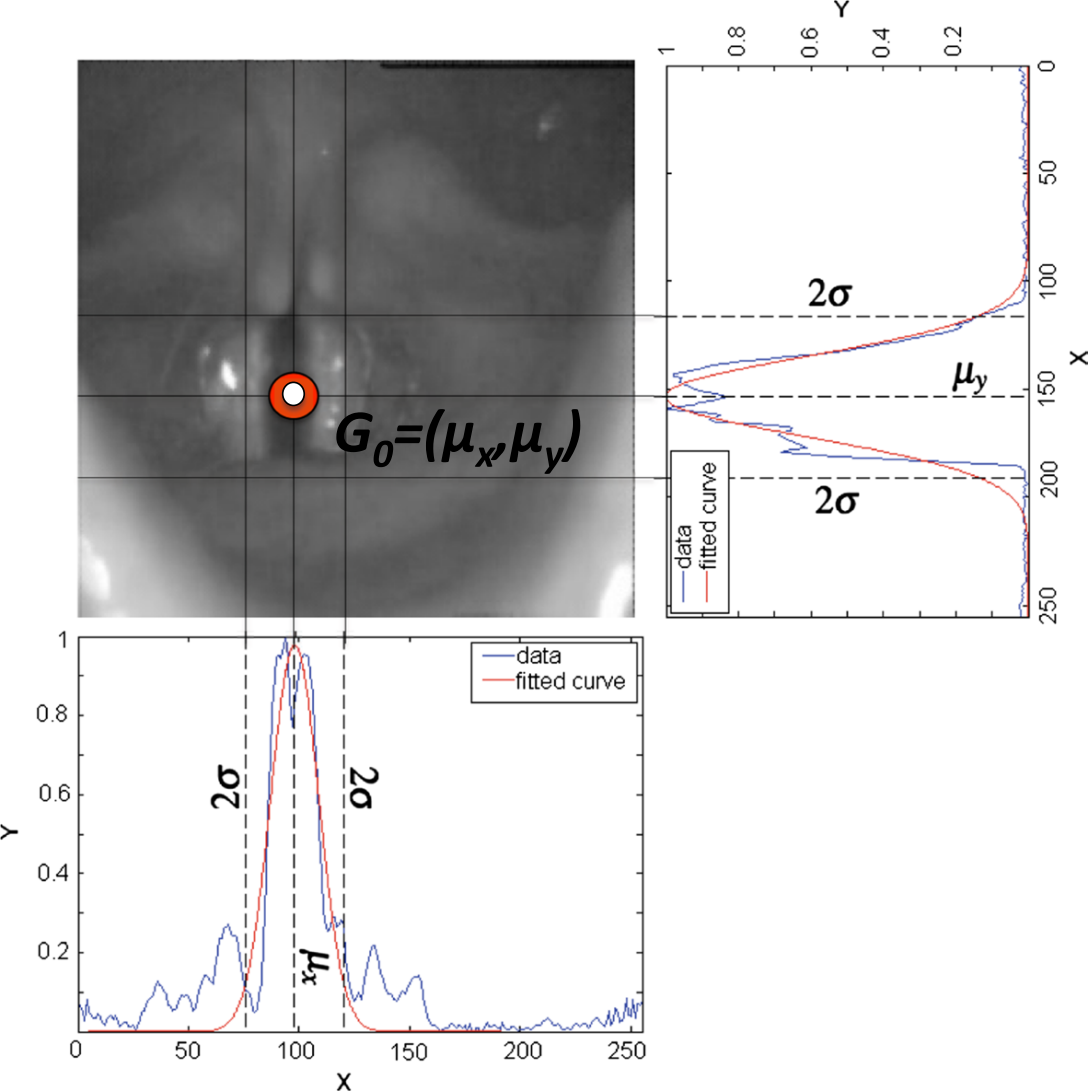
Intersection between $TIV_{c}$ and $TIV_{r}$ means. The peaks of the $TIV_c$ and $TIV_r$ are intersected in order to obtain an approximation of the glottal center, this point is used as a initial feature to compute the glottal main axis

One of the most widespread representations of the vibratory pattern of the vocal folds in the temporal domain is the VKG. The VKG captures the vibration of the vocal folds in a single line placed in a specific location of the glottal area taking as reference the glottal main axis (*g*). The choice of this specific location affects the results of the analysis, but in most of the applications the preferred choice is a line placed in the central part of the glottis in order to get the maximum glottal width amplitude ($g_{max}$). Subsequent works showed that on average, the optimum position is close to the middle of the visible length of the vocal folds ($g_{max}=$ 43.8, 41.1 and 46.5 %) [5]. Additionally to the central line, it is important to perform a multi-line VKG [40] to detect movements alongside the glottal axis *g* at discrete positions ($g=$ 1, 2, 3,..., 99, 100 %). The location of the main axis is usually obtained by manual intervention, glottis segmentation or after performing a PVG. The length of *g* is given by the posterior *P* and anterior *A* endings of the visible anterior-posterior glottal axis. Hence, *P* corresponds to $g=$ 1 % and *A* corresponds to $g=$ 100 %. The main concern with VKG is in the difficulties of obtaining a reliable scanning of the same location of the anatomical structure due to the endoscope displacements and the wrong assumption of a vertical orientation of the glottis. In order to solve these limitations, the information obtained by the ROI can be used to compensate both the endoscope motion and the slope of the glottis. First, it is necessary to fit the model that better adjusts to the glottis data (points inside the glottal gap). Nonetheless, such points are not available until the segmentation is performed, being impossible to predict the *g* axis. Based on the mean intersection between $TIV_c$ and $TIV_r$ (see Fig. 7) it is possible to obtain a good approximation of one of these points, the glottal center ($G_0$) (9). Under the assumption that *g* is a straight line, the set of possible *g* would be all the lines crossing $G_0$. The model selected for *g* is a linear function $h_{\theta }$ (10) with parameters $\theta _0$ and $\theta _1$ that are to be minimized applying a specific cost function *J* (11). The minimization procedure for finding *g* is described in 12.

9 $$\begin{aligned} G_0=(\mu _x,\mu _y) \end{aligned}$$

10 $$\begin{aligned} hypothesis: h_{\theta }(x)=\theta _0+\theta _1x \end{aligned}$$

11 $$\begin{aligned} J(\theta _0,\theta _1)=\frac{\sum \nolimits _{x=1}^{X_{cr}-X_{cl}}I_{{ROI}_{t=min}}(x,h_{\theta })}{X_{cr}-X_{cl}} \end{aligned}$$

12 $$\begin{aligned} g(\theta _0,\theta _1)=\underset{\theta _0,\theta _1}{\text {argmin}}\,\,J(\theta _0,\theta _1) \end{aligned}$$

**Fig. 8:**
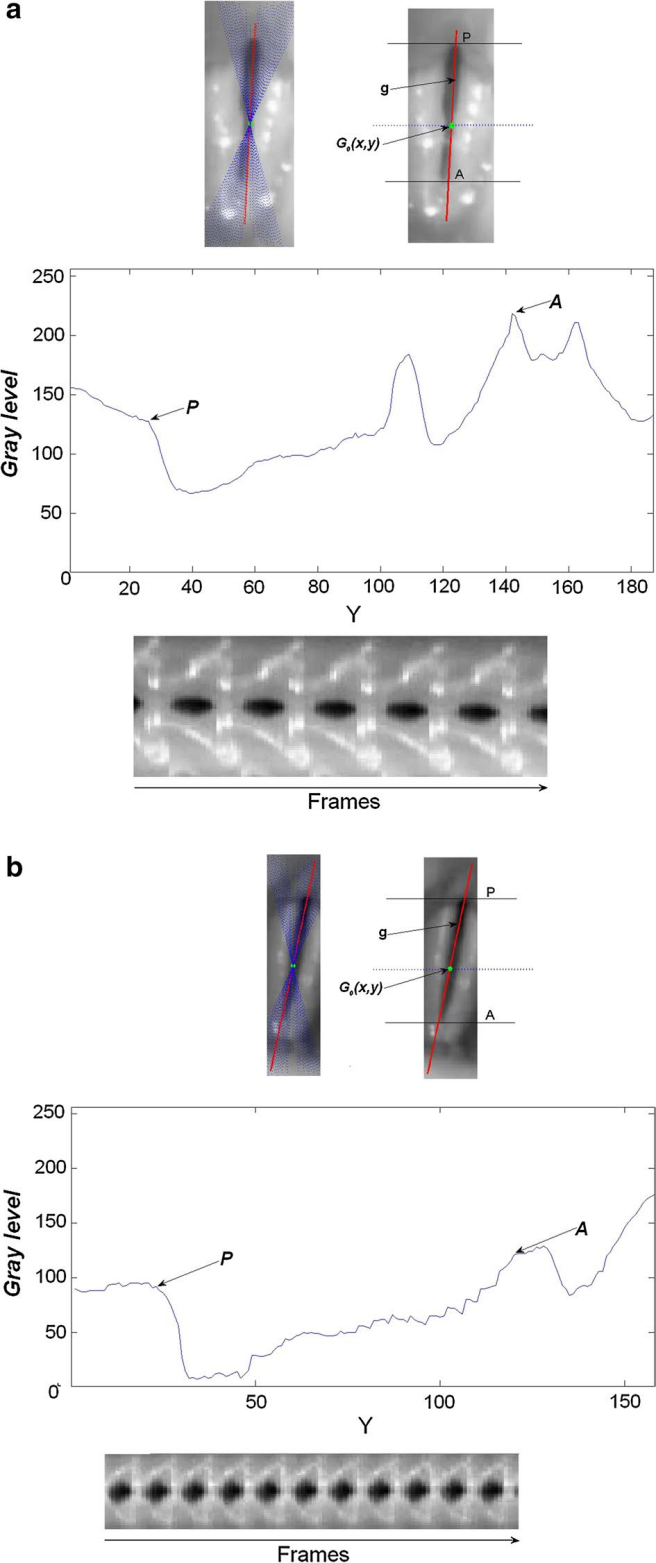
Automatic glottal main axis detection. **a**, **b** Show the complete procedure to get *g* in 2 HSDI. *Top* Glottal main axis, drawing in *red* and all the possible candidate *lines* in *blue*; *Middle* Intensity profile of the glottal main axis; *Bottom* VKG of the compensated ROI

$g(\theta _0,\theta _1)$ can be understood as the line in which the sum of all of its elements has the lowest intensity (see red plot in top of the Fig. 8). The parameter $\alpha$ defines the angle of *g* with respect to the $x-axis$ and it is used to vertically orientate the glottal main axis (13).

13 $$\begin{aligned} \alpha =\arctan (\theta _1) \end{aligned}$$

The graph of the middle row in Fig. 8 represents the intensity profile of *g*. The points with abrupt changes of intensity are candidates to be anterior or posterior points of the glottis. The two most external points that do not correspond to the ROI boundaries are chosen. The high peaks occurring between these extrema can be understood as a discontinuity in the glottis (e.g. the glottal area is split by the presence of nodules or other artifacts). These anterior and posterior points represent $g=$ 1 % and $g=$ 100 % respectively, the bottom part of the Fig. 8 shows the final VKG including the endoscope motion and rotation compensation drawn without user intervention.

## Results

### Database

In order to demonstrate the strengths and limitations of the proposed method, several experiments are carried out using 22 videos from the database provided by Dr. Erkki Bianco and Gilles Degottex [41]. The HSV system used to record the sequences is a Richard Wolf—ENDOCAM 5562. The vocal folds are filmed through a rigid endoscope which passes through the mouth, connected to a high-speed camera providing 4000 colored images per second with a resolution of 256 × 256 pixels. However, the actual sampling rate of the video sequences available in the database is 6665 img/sec, since the used MPEG-2 video codec resampled the sequences. The distance between the head of the camera in the oropharynx and the vocal folds is variable. The database includes usual phonatory modes as Mode I (the most common phonatory), mode II (usually present in a high-pitched voice) and particular phonatory situations such as: breathy voice, tense voice, pressed voice exhaled and inhaled fry. The videos present different illumination levels, contrast, partial occlusion of the glottis and lateral displacements of the camera. Some of them correspond to patients with nodules in the vocal folds.

### Pre-processing adjustment

**Fig. 9:**
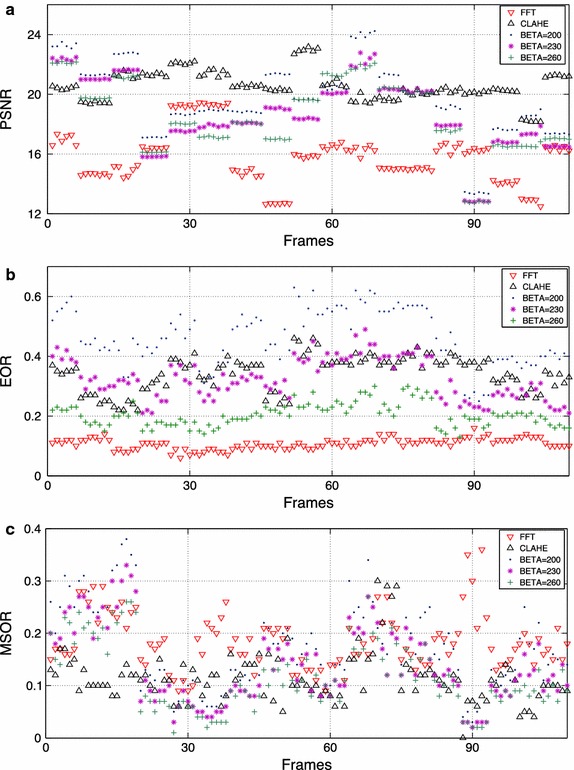
Comparison of the pre-processing algorithms. The objective evaluations employed 110 HSDI images, extracted from the 22 videos of the database: **a** The PSNR graph; **b** EOR graph; **c** MSOR graph

**Fig. 10:**
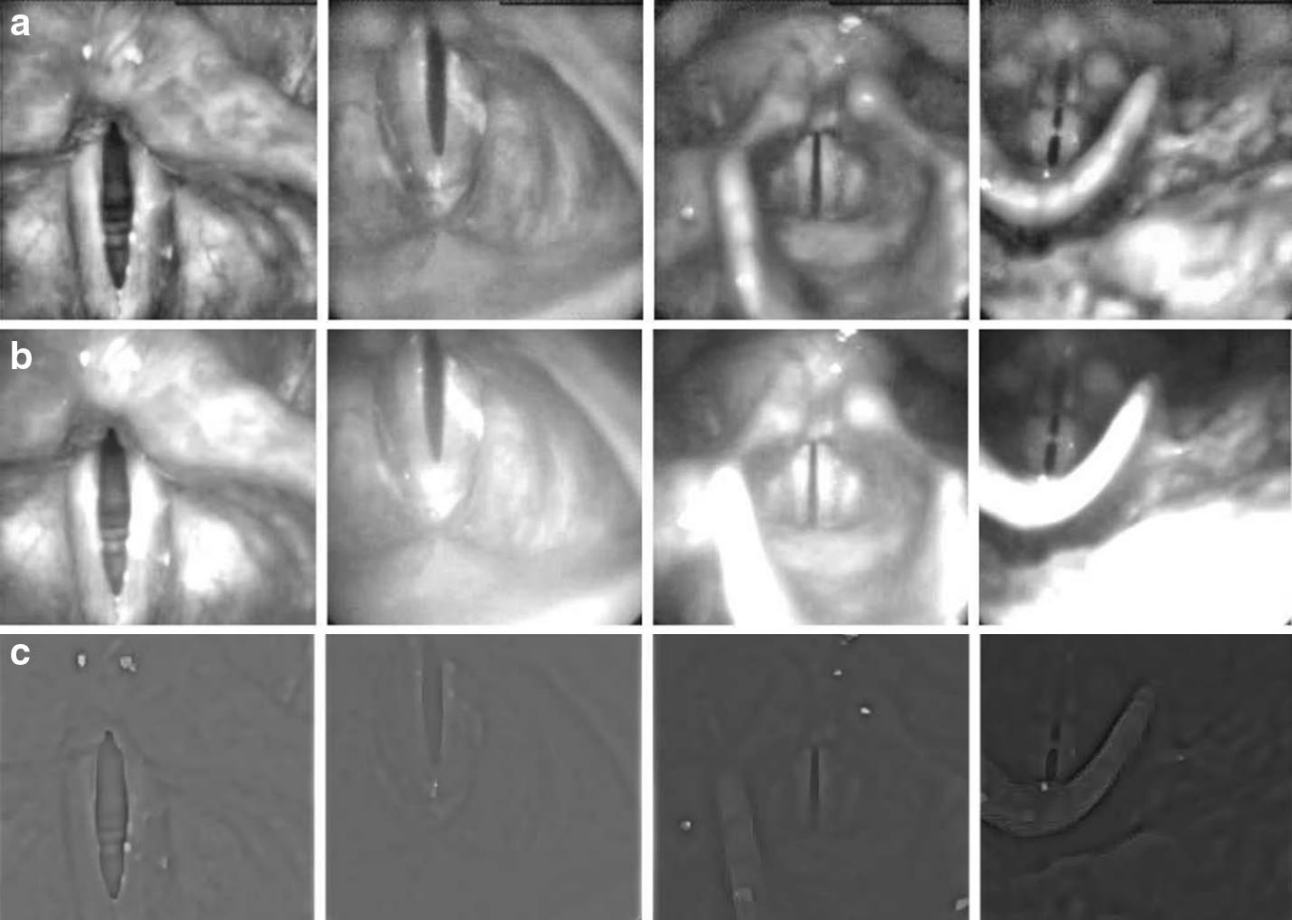
Subjective evaluation of the pre-processing algorithms. A visual representation of the different enhancement methods for four different HSDI. **a** CLAHE; **b** non-linear transformation with $\beta =200$; **c** FFT enhancement

Before validating the reliability of the algorithm some parameters have to be adjusted and some justifications need to be done. Firstly, it is necessary to justify the selection of the enhancement method considering subjective and objective criteria. The quality of the image enhancement techniques is difficult to assess, since evaluating enhancement techniques is still an open problem. The goal of the enhancement is to improve the contrast and illumination of the image, allowing a machine-based vision analysis. Some of the objective measures used for evaluating the enhancement method are Mean Square Error (MSE) and Peak Signal-to-Noise-Ratio (PSNR). However, they are not suitable for many applications and fail to accurately reflect the subtleties of human perception. In [42] an interesting framework is proposed combining three measures including PSNR, Edge Overlapping Ratio (EOR) and Mean Segment Overlapping Ratio (MSOR), corresponding to three image features including intensity, edge and segment. In order to evaluate the performance of the enhancement methods to the problem under study, the objective measure proposed in [42] is employed to 110 HSDI, extracted from the 22 videos of the database. Considering the literature, three enhancement methods are used; FFT, CLAHE and non linear transformation. The non linear transformation is tested with different values of $\beta$ with an incremental step of 30 from 100 up to 300. The obtained results are presented in Fig. 9 and summarized in Table 1 for the most relevant cases. The first graphic describes the intensity changes before and after enhancement (PSNR); the second describes the similarity between edges; and, lastly, MSOR describes the similarity between regions. For laryngeal HSDI, well defined edges and well delimited regions (EOR, MSOR) should be prioritized to facilitate the latter segmentation step. After analyzing the objective results and considering also the subjective evaluation based on visual inspection of the image contrast (Fig. 10) and specially the reduction of the flashing effect, the non-linear transformation with parameter $\beta =200$ is chosen because it keeps a good balance between objective and subjective evaluations.

**Table 1:** Summary of the results reported in Fig. 14 for the different image enhancement techniques used

	FFT	CLAHE	β = 140	$\beta$ = 170	$\beta$ = 200	$\beta$ = 230	$\beta$ = 260
PSNR	15.80	20.57	30.06	24.55	19.66	18.69	18.52
EOR	0.11	0.34	0.59	0.51	0.46	0.32	0.20
MSOR	0.18	0.11	0.11	0.13	0.17	0.14	0.12

### ROI adjustment

The ROI permits to reduce the number of false detections, and is adaptively updated to be tolerant for camera displacements. Firstly, it is necessary to determine $TI_{X}$ and $TI_{Y}$, analyzing the overlapping between the $TIV_c$ and $TIV_r$ curves from the HSDI sequence. The coefficients that regulate the cut-off points are: $\kappa _x$ and $\kappa _y$. Both coefficients can be set indifferently, depending on whether the ROI is to be more compressing in the *x* or *y* axis. For instance a *TI* of 99.7 % ($\mu \pm 3\sigma$) would include non-relevant information to the ROI. Conversely, if *TI* is set to 68 % ($\mu \pm \sigma$) the ROI would over-adjust to the glottis area, causing loss of information. Based on the experimentation around the 22 HSDI sequences available in the database and considering no loss of information, a good tradeoff to fix $\kappa _x$ and $\kappa _y$ is in the range [2, 3]. In this paper both coefficients were fixed with a value of 2.

**Fig. 11:**
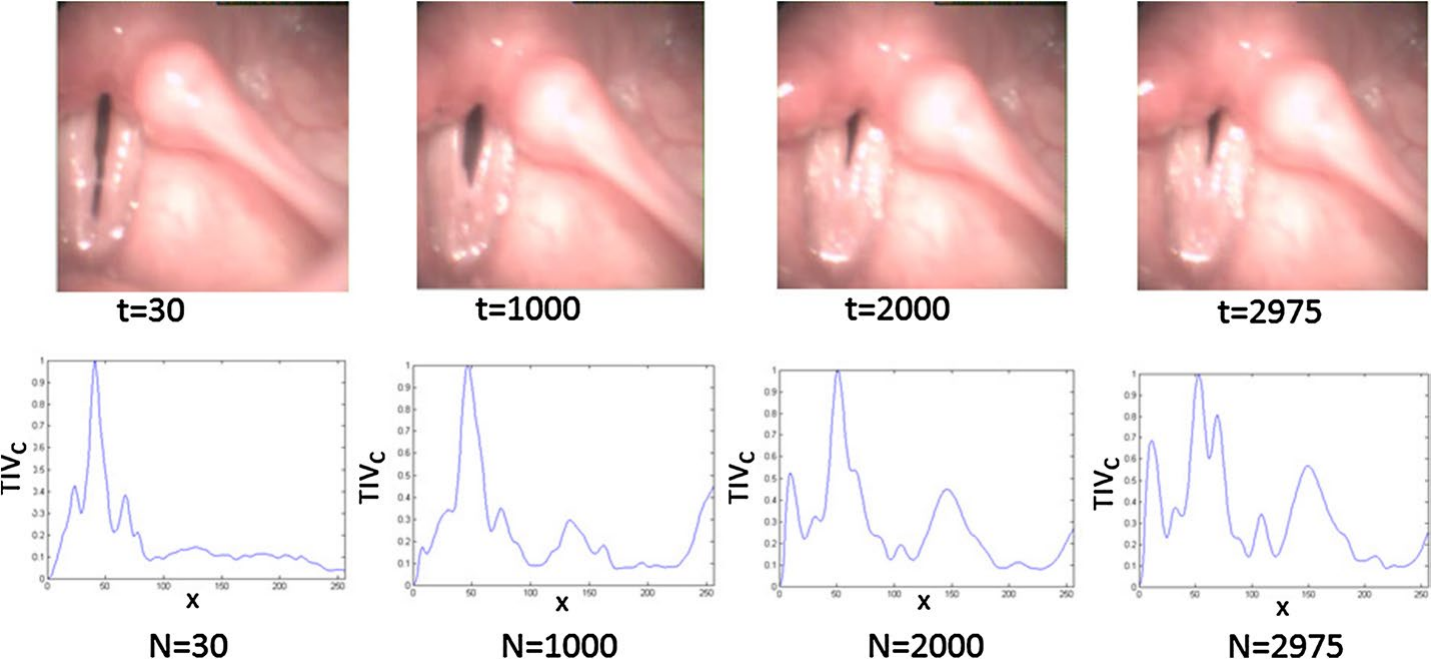
Effect of the transversal motion in $TIV_{c}$ The importance of recomputing the ROI is illustrated plotting different $TIV_c$ for different values of *N*: $N=30$, $N=1000$, $N=2000$, and $N=2975$

The parameter *N* is essential to tolerate the camera displacements. In order to demonstrate this fact, Fig. 11 shows 4 frames in different instants of the glottal cycle with their corresponding $TIV_c$ plots. The instants of time under consideration are: $t=$ 30, 1000, 2000, 2975. It is possible to check by simple inspection, that increasing *N* deviates $TIV_c$ from the gaussian pattern. The explanation to this phenomenon is related with the horizontal motion of the camera during the recording. This causes additional peaks that do not belong to the ones produced by the vocal folds motion, to produce an erroneous gaussian fitting and to generate wrong cutoffs elections. An important conclusion obtained from these examples is referred to the average position of the glottis: the lobe with the maximum peak in $TIV_c$ will be the average position of the glottal gap.

**Fig. 12:**
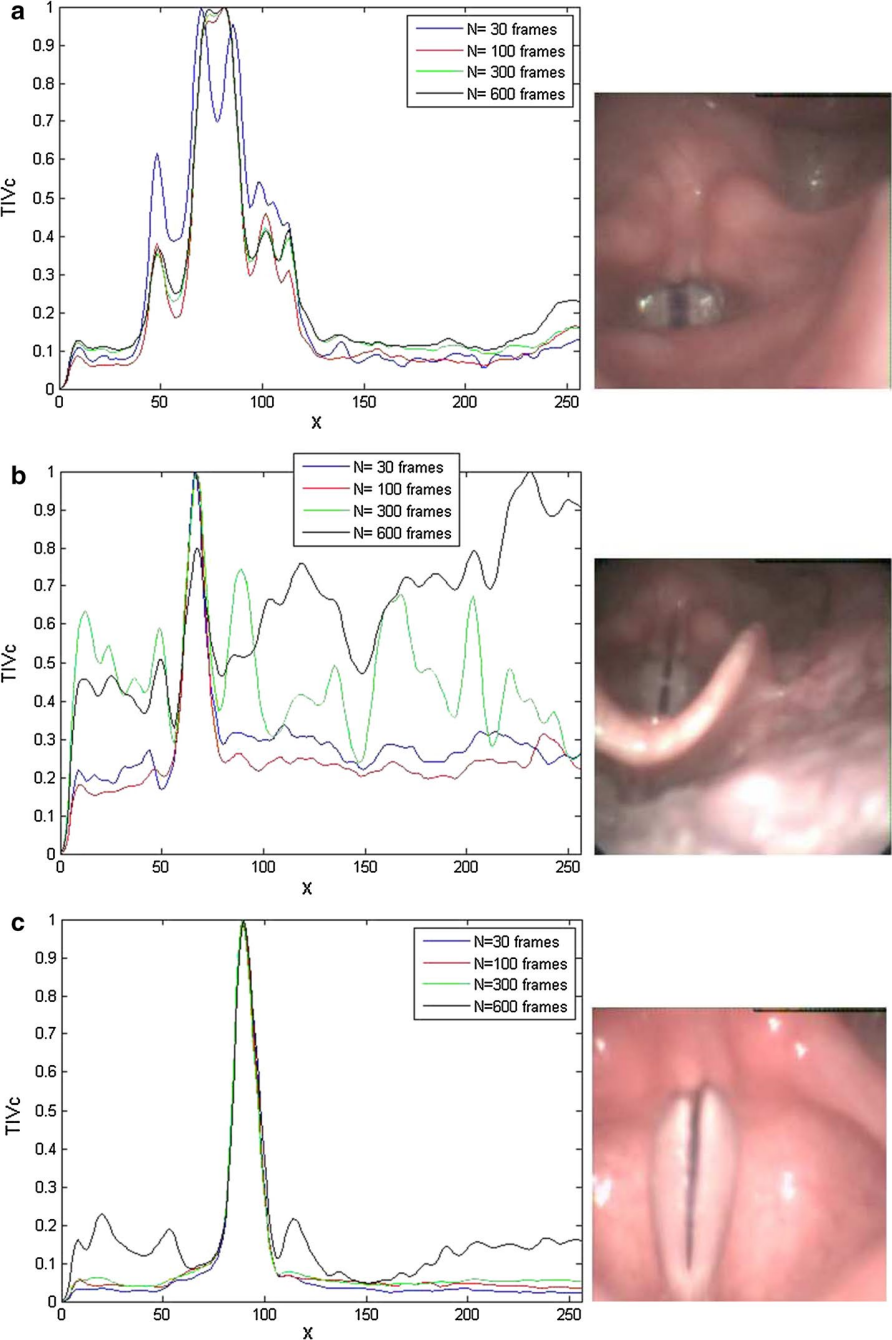
Evaluation of the effect of different. *N* settings. Graphical representation of the variation of *N* for three different HSDI sequences. $TIV_c$ for $N=30$ (*blue line*), $N=100$(*red line*), $N=300$ (*green line*) and $N=600$ (*black line*)

Through experimentation, it is observed that the minimum *N* to achieve a robust ROI is that containing at least one complete glottal cycle. For instance, with a high-speed data rate ($HSD_{rate}$) of 6665 frames per second (0.15 ms per frame), and a fundamental frequency of phonation of $\sim$236 Hz (period equal to 4.23 ms) the minimum value of *N* to be chosen is approximately 28. Figure  12 shows three different images belonging to three different HSDI recordings, each of them with their respective $TIV_c$ plots. The plots present a high complexity due to the presence of occlusions caused by the laryngeal structures. However this fact does not affect the proposed procedure, since this works analyses the average intensity position of the glottal gap for a set of *N* consecutive frames. In Fig. 12a, with $N=30$, there are two peaks that represent the two vocal folds, and the valley in the middle is the glottal space during the opening phase of the glottal cycle, meaning that a complete cycle has not been reached ($\sim$41 frames per cycle). Meanwhile with $N=100$ (red plot) a complete glottal cycle is included, and the width of the Gaussian completely covers the glottis. Increasing *N* leads to many fluctuations close to the maximum peak. Figure 12b ($\sim$38 frames per cycle) is even more demanding because there is a significant occlusion and the glottal gap is not clearly visible. Nonetheless, due the minimal presence of lateral movements, the maximum peak is conserved for all the different values of *N*, but in concordance with the increase of the number of frames, $TIV_c$ starts losing its Gaussian-like shape. Figure.12c ($\sim$17 frames per cycle) represents an ideal case without camera movements, in which the performance of the algorithm is not affected by the choice of *N*. The optimal *N* is in the range between one glottal cycle ($Gc_o$14) and one motion cycle of the endoscope ($Mc_e$15).

14 $$\begin{aligned} Gc_o= T_o/HSD_{rate} \end{aligned}$$

15 $$\begin{aligned} Mc_e=T_e/HSD_{rate} \end{aligned}$$

16 $$\begin{aligned} N \in {[GC_o,Mc_e]} \end{aligned}$$

### ROI based on intensity variation vs ROI based on morphological operation

It is hard to decide which method is the best for detecting the ROI, and even harder to compare the performance between them, since all have been evaluated with different databases. However, in order to provide objective results the steps described in [18] are implemented and compared with the proposal. The main reason for choosing [18] for comparison was based on the fact that this procedure is also fully automatic, and the framework followed to solve the segmentation presents some similarities with this work.

**Fig. 13:**
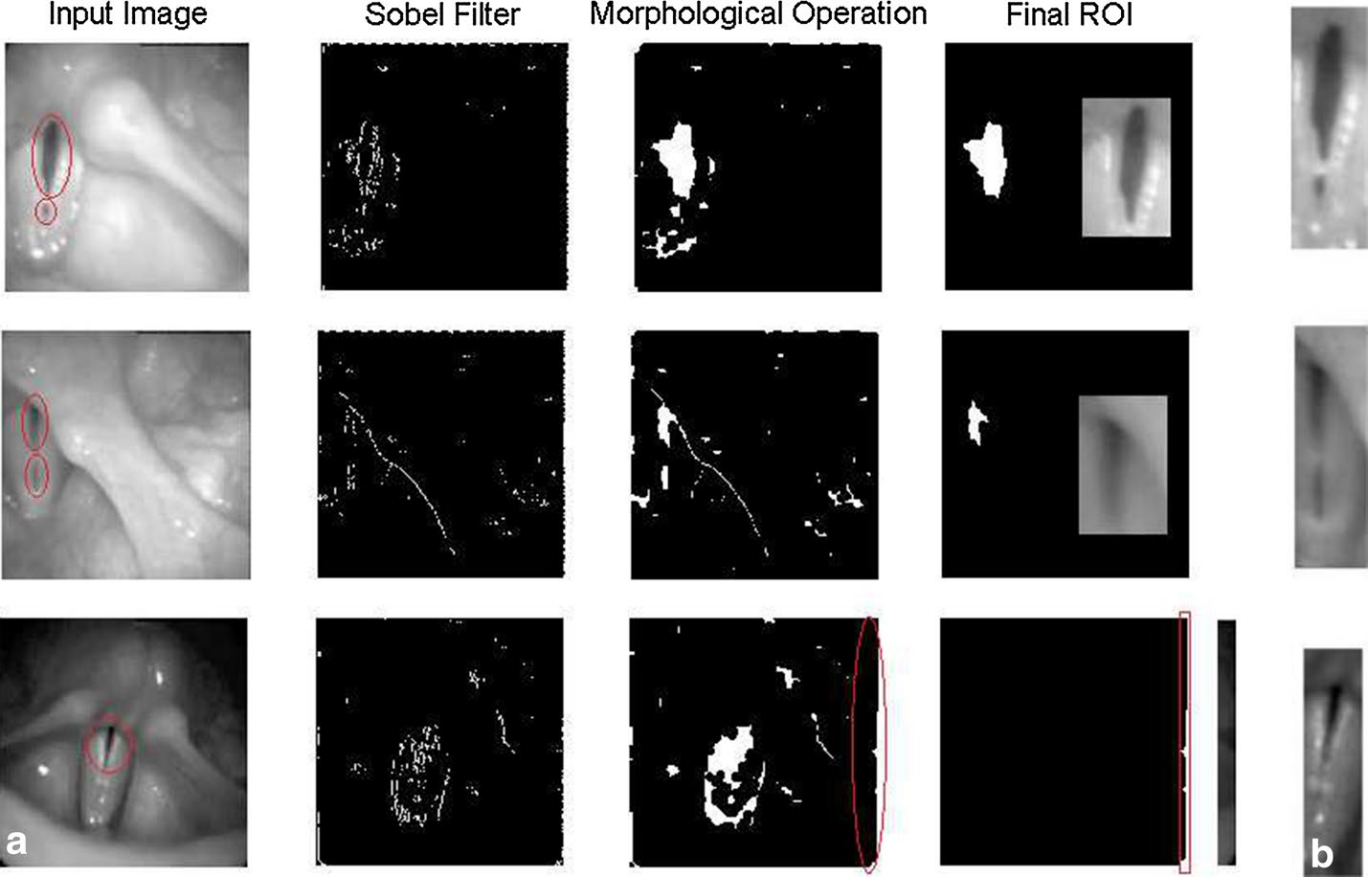
ROI detection using two approaches. **a** ROI detection according to [18]; **b** Final ROI obtained using the proposed approach

An in depth analysis of the algorithm proposed in [18] lets identify two aspects that need further research to improve the results. The first one is related with choosing the area in the landmarks, since in pathological cases the glottis might be divided in two parts, and the algorithm might identify only one of them. This is depicted in the first and second row of Fig. 13a. In both landmarks the glottis is split into two parts, which means that one of the regions will be discarded leading to an erroneous segmentation. The second drawback occurs when there are large artifacts with vertical orientation, like those depicted in the third row of Fig. 13a. These artifacts are common due to reflections of the light inside the tube of the endoscope that carries the camera. Contrariwise, the methodology proposed for ROI detection solves the first problem computing the maximal intensity variation of a region, which means that it is not affected by glottis splitting. Regarding the second drawback; not false detections is produced since there are not change in the intensity along time. The algorithm proposed in this paper for the ROI detection is based on intensity variation, so in those cases with no movement of the vocal folds the algorithm will fail. As a matter of comparison with respect to the approach found in [18], the images showed in Fig. 13b were obtained using the intensity variation approach proposed in this paper.

### Comparison among manual and automatic segmentation

One of the limitations usually found while segmenting images, is that there is no established ground truth about the boundaries to be detected. In others words if an image is presented to ten different people and they are instructed to perform a manual segmentation, most probably they end up with ten different results. This universal problem is even more significant during the segmentation of the glottal gap, since in addition to the problem of the ground truth, there is no standard metrics to evaluate the distinct algorithms. Additionally there is no common database that could be used to compare the results. To mitigate these limitations, the literature refers different metrics that have been used to evaluate the results: DICE and an area error [17]; a multipoint scale comparison [18]; and other approaches like in [26] that choose some points of interest and follow them along time. Another common alternative is to show some of the most relevant glottis delineations for an visual inspection of the performance [25].

**Fig. 14:**
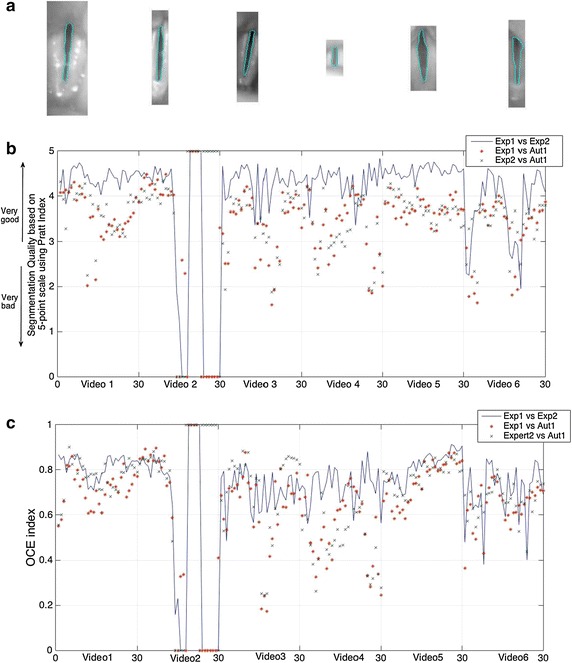
Summary of the experimental results. *Blue line* (Exp1 vs. Exp2) baseline, *red asterisk* (Exp1 vs. Aut1), *green cross* (Exp2 vs. Aut1). **a** HSDI subset; **b** PI of the comparison between*Exp1*, *Exp2* and *Aut1*; **c** Resulting OCE of the comparison between *Exp1*, *Exp2* and *Aut1*

In this work, the experimental evaluation followed consists on evaluating the similarity between two manual segmentations carried out by two different experts *Exp1* and *Exp2*, the segmentation obtained with the algorithm reported in [18] *Aut2*, and the proposed method *Aut1*. All the videos belonging to the database were evaluated; however, for the sake of simplicity, only the results corresponding to 6 video sequences are presented. The criterion for choosing the subset of videos is based primarily on finding those that present the most demanding and challenging scenarios, such as presence of laryngeal structures, camera movements, brightness problems, depth differences, differences in levels of intensity, and closure defects of the vocal folds (Fig. 14a). In this way, the tests are carried out in conditions that are expected to be similar to those found in the clinical environment. The first trial compares the segmentations using the Pratt index (PI) [43]. The PI computes a figure of merit that measures the similarity between boundaries, where 1 indicates that the two edges are equal, and 0 that there is not similarity at all. For the sake of simplicity and better visualization of the results, the quality of the segmentation is analyzed using a 5-point scale directly linked with the PI. The second test repeats the same procedure but using the object-level consistency error (OCE) [44] as an objective metric. The OCE quantifies the similarity or discrepancy between a segmented image and the image considered as ground truth at the object level, taking into account the existence, size position and shape of each fragment, while penalizing both over-segmentation and under-segmentation. The plots in Fig. 14b, c show the results obtained for the 30 first frames after comparing manual segmentation (*Exp1*) and (*Exp2*) and the proposed method (*Aut1*) using PI and OCE respectively. As expected, *Exp1* vs *Exp2* presents the best results in most of the videos. However in video 2, there are many values with PI and OCE equal to zero. This is because the two experts do not coincide in the definition of the closing phase, since in some frames one expert decided not to draw any boundary while the other did. The decision of drawing a boundary is a subjective task that depends on each person’s expertise. The difference between PI and OCE when comparing manual and automatic segmentations is lower that the one obtained among the experts, this may be caused by the criteria used to determine whether a pixel belongs to a boundary or not. The manual segmentation depends on the expert’s skills to differentiate the various levels of gray, while the automatic methods use standard criteria to merge regions. Some minors over-segmentation appear when the glottis is split in two regions separated by a few pixels. This is originated by the refinement step (active contour merging), since the two active contours found in both regions merge into one when they get in contact. The final results of the 6 videos are summarized in Table 2, taking as baseline the results of *Exp1* vs *Exp2*.

**Table 2:** Summary of the comparison obtained between *Exp1*, *Exp2*, *Aut1* and *Aut2*, using OCE and PI figures of merit

	Video1			Video2			Video3		
	*Exp1*	*Exp2*	*Aut2*	*Exp1*	*Exp2*	*Aut2*	*Exp1*	*Exp2*	*Aut2*
PRATT									
* Exp2*	*0.88*	–	–	*0.67*	–	–	*0.85*	–	–
* Aut1*	0.72	0.74	0.56	0.63	0.48	0.24	0.67	0.67	0.59
* Aut2*	0.44	0.47	–	0.26	0.26	–	0.69	0.68	–
OCE									
* Exp2*	*0.81*	–	–	*0.59*	–	–	*0.71*	–	–
* Aut1*	0.72	0.77	0.57	0.51	0.43	0.15	0.61	0.67	0.51
* Aut2*	0.44	0.46	–	0.16	0.18	–	0.57	0.61	–

	Video4			Video5			Video6		
	*Exp1*	*Exp2*	*Aut2*	*Exp1*	*Exp2*	*Aut2*	*Exp1*	*Exp2*	*Aut2*
PRATT									
* Exp2*	*0.84*	–	–	0.76	–	–	*0.77*	–	–
* Aut1*	0.58	0.53	0.20	*0.77*	*0.77*	0.72	0.57	0.61	0
* Aut2*	0.21	0.18	–	0.60	0.61	–	0	0	–
OCE									
* Exp2*	*0.72*	–	–	0.71	–	–	*0.68*	–	–
* Aut1*	0.48	0.43	0.17	0.74	*0.78*	0.68	0.54	0.57	0
* Aut2*	0.14	0.11	–	0.53	0.55	–	0	0	–

The best results are mentioned with a italic values for comparison purposes

The framework proposed is implemented in Matlab with a computation cost of 0.58 s/image, tested in a personal computer with 2.4 GHz Intel core i5 processor and RAM memory of 8 GB. Other automatic techniques report times of: 3.52 s/image with an implementation in C++ by using a Pentium IV-3GHz with 1 GB of RAM [20]; and 1.81 (s/image) for the first and 2.17 (s/image) for the second video set with an implementation in Matlab by using a dualcore Intel 2.67 Ghz processor with 8 GB RAM [17]. Semi-automatic techniques such as [26] and [27] report times of 0.01 s/image and 0.04 s/image respectively without considering the user intervention. Since additional implementation details are not given and these algorithms were implemented using different programming languages, a detailed comparison with them is difficult.

## Discussions

In order to objectively compare the results under similar conditions, the automatic algorithm presented in [18] and two manual delineations are used as baselines for comparison. In order to assess the accuracy of the segmentations two objective metrics are implemented (PI, OCE). The different trials are summarized in Table 2 and in Fig. 14. As it was expected, the best accuracy is obtained when both manual segmentations are compared, even so the values of the metrics do not reach the 90 % of similarity, which proves the complexity of the addressing problem and also shows the lack of concordance between ground-truths. The second best results occur when the proposal is compared against the manual segmentations. The accuracy in these cases is 10 % lower than the best one but 10 % higher than [18] on average. The objective evaluation and comparison of the segmentation algorithms for the glottis detection would require not only common metrics but also publicly accessible databases annotated with manual segmentations. This would be the first step so the researchers in the field have an objective method to evaluate their algorithms.

The results show the benefits of using an adaptive ROI calculated using temporal information in comparison with an approach based on morphological operations, demonstrating a good accuracy even in the most demanding cases (e.g. glottis with partial occlusion, videos with camera movements, inter video depth differences, presence of nodules producing an inappropriate closure of the vocal folds), and reliability against external artifacts introduced by intrinsic recording problems. The performance, effectiveness and validation of the approach is also demonstrated in different phonatory situations; modes I and II of phonation, breathy voices, tense voices, pressed voices, and exhaled and inhaled fry.

Despite of the advantages of the proposed method, there are two main issues that need to be addressed in future works: the empirical selection of the template, and the identification of the ROI during the phonation onset. The first one occurs when the glottal opening increases its size abruptly since the correlation among the template and the previous merging step produces an initialization smaller than the one expected. The solution to this problem could stand on increasing the number of iterations in the post-processing step. The second issue could be addressed by back-propagating the information obtained during the steady phonation to the onset interval.

In Addition, there are some pathological cases that need attention to ensure the generalization of the proposal, namely: polyps, cysts, Reinke’s edemas, leukoplakias and hemorrhagic lesions. In principle, these pathologies should not affect the performance of the algorithm proposed since the presence of these disorders should not modify the intensity along time (ROI detection is not affected) and most of them are presented as white tonalities. However, hemorrhagic lesions could provide dark tonalities that would increase the regions that do not belong to the glottis after the first merging step. Since the correlation merging step is based on the glottal shape, it is expected that all of the regions that do not belong to the glottis will be discarded after this step. These pathological cases should be studied in details to guarantee the generalization of the approach.

## Conclusions

The present work proposes an alternative algorithm to the existing methods for detecting and tracking the glottal gap. The framework consists of a set of modules to pre-process, detect the region of interest, delineate the contours, and refine the glottis shape. The pre-processing step is throughly tested by objective and subjective means in order to obtain the most suitable algorithm. The point-wise non linear transformation algorithm is chosen since it presents the better trade-off between objective and subjective evaluation and also mitigates the influence of flashes which affects the performance of the ROI detection. On the other hand, the ROI detection takes advantage of the temporal intensity information of the HSV, and is adaptively updated every *N* frames according to an extensive evaluation. Thanks to its adaptability, the ROI provides reliability against the camera and/or patient displacement, reduces the influence of false detections, it is robust when the glottis is divided in two or more regions, and is able to manage the presence of glottal chinks when the cut-off points are chosen appropriately. The segmentation module uses the well-known watershed transform with two merging steps based on JND and a template correlation: the first merging step fuses regions based on the sensibility of the human visual system to the changes of luminance; the correlation merging step gives additional information about the position and shape of the glottis which lets differentiate between glottal and non-glottal regions. Finally, to refine the segmentation and solve any problem with the previous steps, a region based active contours modeling is performed. One of the novelties of the proposed algorithm relies on the methods used to identify the ROI, as well as the combination of the watershed transform with a standard template for the merging process. The methods described in this paper have also been applied to propose a new procedure to automatically synthesize multiline VKG by identification of the glottal main axis. Such task can be performed using only the information provided by the adaptive ROI. First, it is necessary to find the glottal gap center in the frame with the maximal glottal opening since that point belongs to the glottal main axis. The glottal center is obtained by the intersection between $TIV_c$ and $TVI_r$ means. Later, all the lines that cross $G_0$ are regarded as main axis candidates, but only the one that minimizes the sum intensity of all of its pixels is selected. The main axis can be used to compensate camera rotations and displacements, leading to a more accurate representation of the VKG. The proposed method permits a fully automatic segmentation procedure in which the parameters that set the algorithm are also adjusted automatically using the statistics obtained from the video sequence and empirical observation.

## Abbreviations

SMS: slow motion stroboscopy
HSDI: high speed digital imaging
VKG: digital video kymogram
GAW: glottal area waveform
PVG: phonovibrograms
ROI: region of interest
FFT: fast Fourier transform
AHE: adaptive histogram equalization
CLHE: contrast limited histogram equalization
CLAHE: contrast limited adaptive histogram equalization
$TIV_{c}$: total intensity variation in columns
$TIV_{r}$: total intensity variation in rows
MAE: mean absolute error
MC: motion compensation
JND: just noticeable difference
MSE: mean square error
PSNR: peak signal-to-noise-ratio
EOR: edge overlapping error
MSOR: mean segment overlapping ratio
OCE: object-level consistency error
